# Niches, Population Structure and Genome Reduction in *Ochrobactrum intermedium*: Clues to Technology-Driven Emergence of Pathogens

**DOI:** 10.1371/journal.pone.0083376

**Published:** 2014-01-17

**Authors:** Fabien Aujoulat, Sara Romano-Bertrand, Agnès Masnou, Hélène Marchandin, Estelle Jumas-Bilak

**Affiliations:** 1 UMR 5119 ECOSYM, Equipe Pathogènes et Environnements, U.F.R. des Sciences Pharmaceutiques et Biologiques, Université Montpellier 1, Montpellier, France; 2 Laboratoire de Bactériologie, Hôpital Arnaud de Villeneuve, Centre Hospitalier Régional Universitaire de Montpellier, Montpellier, France; 3 Laboratoire d'Hygiène hospitalière, Centre Hospitalier Universitaire de Montpellier, Montpellier, France; National University, Costa Rica

## Abstract

*Ochrobactrum intermedium* is considered as an emerging human environmental opportunistic pathogen with mild virulence. The distribution of isolates and sequences described in literature and databases showed frequent association with human beings and polluted environments. As population structures are related to bacterial lifestyles, we investigated by multi-locus approach the genetic structure of a population of 65 isolates representative of the known natural distribution of *O. intermedium*. The population was further surveyed for genome dynamics using pulsed-field gel electrophoresis and genomics. The population displayed a clonal epidemic structure with events of recombination that occurred mainly in clonal complexes. Concerning biogeography, clones were shared by human and environments and were both cosmopolitan and local. The main cosmopolitan clone was genetically and genomically stable, and grouped isolates that all harbored an atypical insertion in the *rrs*. Ubiquitism and stability of this major clone suggested a clonal succes in a particular niche. Events of genomic reduction were detected in the population and the deleted genomic content was described for one isolate. *O. intermedium* displayed allopatric characters associated to a tendancy of genome reduction suggesting a specialization process. Considering its relatedness with *Brucella*, this specialization might be a commitment toward pathogenic life-style that could be driven by technological selective pressure related medical and industrial technologies.

## Introduction

Experimental approaches on type or model strains of specific bacterial pathogens has led to fruitful results that revolutionized knowledge about the molecular mechanisms of infection [1]. By contrast, model-based approaches not fully succeeded for opportunistic pathogens, mostly because they formed heterogeneous populations, i.e., organised in species complexes rather than in true species [2]. It is now widely accepted that population structure and genome content depend on bacterial life-styles and niches [3]–[5]. Moreover, the population structure of environment-borne human opportunistic pathogens (EBOP) suggested that sub-groups of strains in a species could be associated with and perhaps adapted to human beings [6] or to human pathological niches [7], [8]. Therefore, exploring population structure is a prerequisite to the description of life-style, niche adaptation and evolution mechanisms originating in the emergence and pathogenesis of opportunistic pathogens.

The genus *Ochrobactrum* belongs to the family *Brucellaceae* in the class alphaproteobacteria and groups bacteria with versatile life-styles inhabiting various niches and displaying dynamic genomes [9], [10]. In most publications in the field of environmental sciences, *Ochrobactrum* strains are studied for their potential applications in bioremediation [11]–[14] or as plant growth-promoting rhizobacteria [11], [13], [15]. *Ochrobactrum* are considered as emerging human opportunistic pathogens, *Ochrobactrum anthropi* and *Ochrobactrum intermedium* being the two main species that cause infections, mostly in immunocompromised patients [16]. Multilocus sequence typing supported the hypothesis that *O. anthropi* displays a human-associated subpopulation but, as for many other EBOPs, the population structure as well as the reservoir(s) of *O. intermedium* are not precisely defined, impairing the understanding of its potential mechanisms of adaptation to human and thereby, its epidemiology and its pathogenesis. *O. intermedium* displays additional interest due to its phylogenetic relatedness to the genus *Brucella* grouping specific pathogens causing brucellosis, a worldwide anthropozoonosis [17]. Contrarily to *Ochrobactrum*, *Brucella* groups allopatric microorganisms that live in a narrow niche and display stable genomes with reduced size in comparison with *Ochrobactrum* genomes [18].

With the aims to explore the relationships of *O. intermedium* with human and to complete the story about the emergence of pathogenic life-styles among *Brucellaceae*, we studied here the population structure and the genome dynamics in a collection of strains encompassing the range of life-styles and habitats of this species.

## Results

### *O. intermedium* life-style and habitat deciphered to construct a representative collection

In order to learn about the habitat and lifestyle of *O. intermedium*, we undertook a literature review and we screened nucleotide databases in June 2013. We found thirty-nine publications presenting 115 strains or clones with confirmed affiliation to *O. intermedium*. In genetic databases, 56 sequences not associated with a publication matched with the reference sequences used for screening (Table S2). One study reported the isolation of *O. intermedium* whereas the strains belonged to the later described species *O. pseudintermedium* (NR_043756) on the basis of 16S rRNA gene sequencing [19]. Conversely, 13 strains or clones (Tables S1 and S2) that actually belonged to *O. intermedium* were affiliated to other *Ochrobactrum* species, mainly to *O. anthropi*. Misidentifications were especially linked to wrong nomenclature in databases. For instance, the partial 16S rRNA gene sequence of several collection strains (CCUG39736, LMG5446, LMG3306, CCUG1838, CCUG44770) are affiliated to the species *O. anthropi* although these strains were transferred to the species *O. intermedium* based on partial sequence of the *recA* gene [20]. Similarly, partial 16S rRNA gene sequence of the type strain of *O. intermedium* CNS 2–75 ( = LMG3301^T^) (NR_026039) is affiliated to *O. anthropi*.

**Figure 1:**
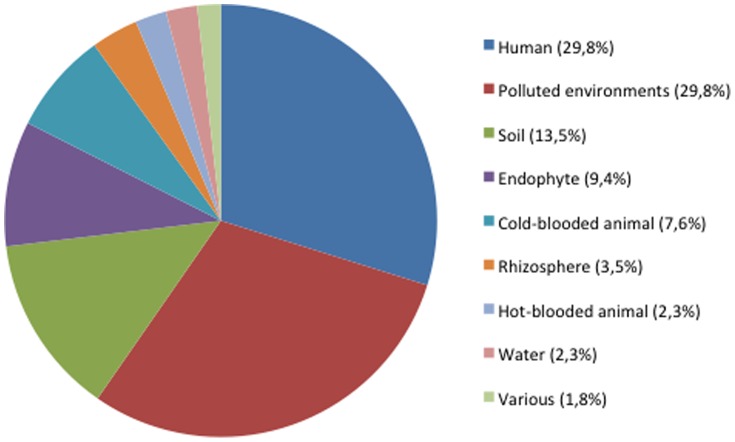
Distribution of the 171 strains (n = 148) and clones (n = 23) of *O. intermedium*/*O. ciceri* identified from the litterature and databases according to the habitat. The figure has been constructed from data presented in Tables S1 and S2.

The databanks survey showed the affiliation of 171 strains and clones from publications (n = 115) and from genetic databases (n = 56) to the species *O. intermedium* on the basis of partial sequence of the 16S rRNA and/or *recA* genes, sometimes completed by phenotypic data. Figure 1 shows their distribution in a wide range of habitats and lifestyles. Strains were found as free-living bacteria or in more or less close associations with eukaryotes for half of the strains (n = 90). *O. intermedium* showed frequent association with human beings (n = 51) and environments polluted by a wide range of compounds (pesticides, herbicides, chromium, cadmium, lead, waste water, oil, petroleum, etc.) (n = 51). *O. intermedium* was also isolated from warm (n = 4) and cold-blooded (n = 13) animals, in association with plants, in the rhizosphere (n = 6) or as endophyte (n = 16) (Table S1). Some plant-associated strains were able to renodulate their host plant and stimulate their growth [15]. The 16S rRNA sequence of *Ochrobactrum ciceri*, a species isolated from plant nodules, displayed 99.8% of similarity with that of *O. intermedium* strain CCUG44770. This species has been described as a new taxon separated from *O. intermedium* mainly on the basis of plant nodules formation (Imran, 2010). Therefore, our database screening approach was not suitable for discriminating *O. intermedium* and *O. ciceri*.

Metagenomic clones matching with the sequences of *O. intermedium* used for database screening were scarce considering the amount of metagenomic data currently deposited in databanks. Only 28 clones could be affiliated to *O. intermedium/O. ciceri*. They were detected in 8 different environments, most of them being technological and polluted environments, such as metal degreasing systems [21], aerosols and fluids in metalworking industry [22], biofouling biofilms, petroleum reservoir, polychlorinated bephenyl (PCB) polluted soil and polyacrylamide (PAM)-degrading consortium. Some clones were detected in velvetleaves seed and only one clone was found in the human skin microbiota (GQ115798) [23].

For available sequences, the presence of an atypical insertion previously described in the 16S rRNA gene [9] is reported in the Table S1. No relationships between insertion detection and habitat or lifestyle could be established.

In this study, we constituted the largest published collection of *O. intermedium*/*O. ciceri* strains (n = 65) (Tables 1 and 2) but mainly focused on clinical isolates. It contained 37 clinical isolates from French hospitals (this work and [9], [10], [16], [24]), 11 environmental isolates (this work and [25]) and to our knowledge all the 17 strains currently available in collections : 12 clinical including the type strains of *O. intermedium* and 5 environmental strains including the type strains of *O. ciceri*. Clinical strains were mainly isolated from the Montpellier Hospital in south of France from 1999 to 2011 but the clinical strain collection was completed with strains from other French towns, countries and continents isolated since 1972. Considering the reservoirs of *O. intermedium*/*O. ciceri* strains and clones in databases, our collection is globally representative of the species diversity except for an underrepresentation of strains from polluted environments. However, the only strain from polluted environment currently available in strain collections (CCUG57381) has been included in the present collection.

**Table 1:** Characteristics of the 49 *O. intermedium* strains of human origin included in this study presented according to MLST results.

Strain	CC	ST	Allelic profiles^a^							46-pb insertion^b^	Origin	Place (town, region or state, country) and date of isolation
			*dnaK*	*recA*	*rpoB*	*aroC*	*omp25*	*trpE*	*gap*			
ADV1	CC68	**68**	11	13	24	18	16	18	15	+	Sputum	Montpellier, Fr, jan 1999
ADV107	CC68	**68**	11	13	24	18	16	18	15	+	Rectum	Montpellier, Fr, feb 2008
ADV32	CC68	**68**	11	13	24	18	16	18	15	+	Rectum	Montpellier, Fr, jan 2003
ADV9	CC68	**68**	11	13	24	18	16	18	15	+	Ear	Montpellier, Fr, aug 1999
LMG5425	CC68	**68**	11	13	24	18	16	18	15	+	Urine	Sheffield, England, before 1983
LMG5426	CC68	**68**	11	13	24	18	16	18	15	+	Urine	Sheffield, England, before 1983
Nim80	CC68	**68**	11	13	24	18	16	18	15	+	Toe nail	Nîmes, Fr, 2006
ADV10	CC74	**69**	10	7	26	20	16	21	18	−	Wound	Montpellier, Fr, nov 1999
ADV101	CC74	**70**	10	7	26	18	16	21	18	−	Rectum	Montpellier, Fr, nov 2007
ADV93	CC74	**70**	10	7	26	18	16	21	18	+	Rectum	Montpellier, Fr, jun 2007
ADV11	CC74	**71**	10	7	26	18	16	21	16	−	Rectum	Montpellier, Fr, nov 1999
ADV24	CC74	**71**	10	7	26	18	16	21	16	−	Axilla	Montpellier, Fr, feb 2002
ADV14	CC74	**72**	10	7	26	20	24	21	16	−	Axilla	Montpellier, Fr, may 2000
ADV56	CC74	**73**	10	7	26	18	24	21	16	−	Rectum	Montpellier, Fr, mar 2005
ADV73A	CC74	**74**	10	7	26	21	16	21	18	−	Rectum	Montpellier, Fr, may 2006
Toul65	CC74	**74**	10	7	26	21	16	21	18	−	Sputum (CF)	Toulouse, Fr, aug 2005
ADV89	S	**75**	12	7	26	18	24	22	19	−	Rectum	Montpellier, Fr, apr 2007
ADV124	CC76	**76**	15	8	23	35	16	26	18	−	Sputum	Montpellier, Fr, nov 2008
CCUG39736	CC76	**77**	15	8	23	31	25	26	18	−	Blood	Umeå, Sweden, 1998
ADV54	CC68	**78**	11	13	24	27	27	18	15	+	Rectum	Montpellier, Fr, sep 2004
CCUG1838	CC68	**79**	22	13	24	18	16	18	15	+	Urine	Göteborg, Sweden, 1972
CCUG44770	CC68	**80**	12	13	24	21	16	21	15	+	Sputum (CF)	Wien, Austria, 2000
LMG379	CC68	**83**	11	13	24	18	28	20	15	+	Ear	Louisiana, USA, before 1988
ADV143B	CC68	**84**	11	13	24	20	16	18	15	+	Rectum	Montpellier, Fr, mar 2010
ADV111	CC68	**85**	11	13	24	21	16	18	15	+	Sputum (CF)	Montpellier, Fr, apr 2008
ADV126*	CC68	**86**	11	13	24	18	22	18	15	+	Axilla	Montpellier, Fr, feb 2009
ADV127*	CC68	**86**	11	13	24	18	22	18	15	+	Axilla	Montpellier, Fr, feb 2009
CRBIP17.121	CC68	**86**	11	13	24	18	22	18	15	+	Peritoneal fluid	Montélimar, Fr, 2005
ADV109	S	**87**	14	15	18	28	19	27	23	+	Blood	Montpellier, Fr, mar 2008
ADV78	CC74	**88**	10	15	26	18	16	21	16	−	Axilla	Montpellier, Fr, oct 2006
ADV42	S	**89**	19	15	27	23	20	28	20	−	Rectum	Montpellier, Fr, nov 2003
ADV67**	CC91	**90**	18	16	20	19	17	31	25	−	Pancreas	Montpellier, Fr, sep 2005
ADV69**	CC91	**90**	18	16	20	19	17	31	25	−	Rectum	Montpellier, Fr, oct 2005
ADV147	CC91	**91**	18	16	20	18	16	31	25	−	Sputum	Montpellier, Fr, sep 2010
LMG3301^T^	CC91	**91**	18	16	20	18	16	31	25	−	Blood	France, before 1988
ADV85	S	**92**	13	16	22	26	24	32	26	−	Rectum	Montpellier, Fr, jan 2007
ADV44	CC93	**93**	12	14	25	24	23	22	19	−	Rectum	Montpellier, Fr, feb 2004
Nim125	CC93	**94**	12	14	25	18	16	22	19	−	Broncho-alveolar lavage fluid	Nîmes, Fr, dec 2008
ADV21	CC96	**95**	16	17	21	21	16	25	21	−	Rectum	Montpellier, Fr, feb 2001
CIP105839	CC96	**96**	16	17	21	29	21	25	21	−	Blood	Pamplona, Spain, before 1998
CIP105840	CC96	**96**	16	17	21	29	21	25	21	−	Blood	Pamplona, Spain, before 1998
ADV35	S	**97**	17	18	17	22	23	23	16	−	Blood	Montpellier, Fr, jun 2003
LMG5446	S	**98**	21	9	28	32	16	24	18	−	Bladder	Georgia, USA, before 1986
Tou55	S	**100**	19	12	16	34	23	30	17	+	Sputum (CF)	Toulouse, Fr, nov 2004
LMG5443	CC68	**101**	11	13	24	30	16	18	15	+	Urine	North Carolina, USA, before 1988
ADV152	S	**104**	24	19	29	18	16	33	27	−	Rectum	Montpellier, Fr, dec 2010
ADV46	S	**105**	23	10	19	25	26	29	24	−	Bladder drain liquid	Montpellier, Fr, may 2004
NAN157	CC74	**135**	12	7	26	21	16	21	28	−	Broncho-alveolar lavage fluid	Nancy, Fr, dec 2010
ADV158	CC74	**136**	15	7	26	33	16	21	28	−	Sinus	Montpellier, Fr, apr 2011

CC, clonal complex; ST, sequence type; Fr, France; USA, United States of America; CF, Cystic Fibrosis. Strains marked by * or ** were isolated in the same patient.

^a^ For each locus, each different allele was assigned an arbitrary number;

^b^ 46-bp atypical insertion described in Teyssier et al. 2003 [9].

**Table 2:** Characteristics of the 15 *O. intermedium* strains and *O. ciceri* type strain of environmental origin presented according to MLST results.

Strain^a^	CC	ST	Allelic profiles^b^							46-pb insertion^c^	Origin	Place (town, region or state, country) and date of isolation*^e^*
			*dnaK*	*recA*	*rpoB*	*aroC*	*omp25*	*trpE*	*gap*			
RT148-2	CC68	**68**	11	13	24	18	16	18	15	+	Water (lake)	Liausson, Fr, 2010
RT148-1P	CC74	**70**	10	7	26	18	16	21	18	−	Water (lake)	Liausson, Fr, 2010
RT168-1	CC74	**71**	10	7	26	18	16	21	16	−	Water (river)	Montpellier, Fr, 2010
FRG10/sat	CC74	**74**	10	7	26	21	16	21	18	−	Nematode (*Heterorhabditis indica*)	Guadeloupe, Fr, 2005
LMG18956 ( = OiC8-6)	CC74	**74**	10	7	26	21	16	21	18	−	Agricultural soil	Grignon, Fr, before 1996
LMG3306	CC74	**74**	10	7	26	21	16	21	18	−	Soil	France, before 1988
RT172	CC76	**76**	15	8	23	35	16	26	18	−	Sand (beach)	Saint Pierre, La Réunion, Fr, 2011
RT190-1	CC76	**76**	15	8	23	35	16	26	18	−	Water (river)	Montpellier, Fr, 2011
CCUG57381	CC76	**77**	15	8	23	31	25	26	18	−	Water from antibiotic production mixed with sewage	Hyderabad, India, 2007
FRG14/sat	CC68	**81**	11	13	24	18	15	18	15	+	Nematode (*Heterorhabditis indica*)	Guadeloupe, Fr, 2005
JLJ57	CC68	**82**	11	13	24	18	24	18	15	+	Pharmaceutical water	Montpellier, Fr, 2005
DO07/sat	CC68	**84**	11	13	24	20	16	18	15	+	Nematode (*Heterorhabditis indica*)	Dominican Republic, 1996
PR17/sat	CC68	**84**	11	13	24	20	16	18	15	+	Nematode (*Heterorhabditis indica*)	Puerto Rico, 1996
CCM7036	S	**99**	20	11	15	29	18	19	22	+	Insect (*Phlebotomus duboscqi*)	Czech Republic, before 2002
RT23-4	CC93	**102**	12	14	25	33	24	22	19	−	Water and sediments (river)	Blois, Fr, 2009
*O. ciceri* DSM22292^T^	CC68	**113**	11	13	24	18	48	18	15	+	Root nodules of *Cicer arietinum*	Faisalabad, Pakistan, 1996

CC, clonal complex; ST, sequence type; Fr, France.

^a^ Strains noted RT were isolated from systematic searching of *O. intermedium* in 200 soil and water samples randomly collected worldwide.

^b^ For each locus, each different allele was assigned an arbitrary number.

^c^ 46-bp atypical insertion in *rrs* described by Teyssier et al., 2003 [9].

### Multilocus genetics

Among the 65 strains studied, MLSA showed a total of 227 single nucleotide polymorphisms (SNPs) in the 7 loci corresponding to 3.6% and 9.5% of polymorphic sites depending on the gene (Table 3). The mean genetic diversity (H) among strains was 0.7795+/−0.0253 and the genetic diversity at each locus (h) is given in Table 3. H in the clinical strain population (0.7936+/−0.0271) did not differ significantly from H in the environmental population (0.7452+/−0.0259), *P* = 0.456. Genes had equivalent mol% G+C contents from 56.6% to 61.7% with a mean value of 59.7% that was similar to the mean mol% G+C contents of the *O. intermedium* chromosomes (57.7%) (http://www.ncbi.nlm.nih.gov/genome/2167?project_id=55963). The number of alleles ranged from 13 (*recA*) to 18 (*aroC*) (Table 3) and did not depend on the size of the sequence studied. The locus *omp25* that codes for an antigenic surface protein displayed a number of alleles (15 against 15.1 on average) and a percentage of polymorphic sites (6.9 against 6.5 on average) similar to other loci. However, this locus had the lowest genetic diversity (0.6322). Indeed, 60% (39/65) of strains shared the allele 16 for this locus and belonged to different CCs or were singleton (Tables 1 and 2). The same particularity was observed for the allele 18 of *aroC* shared by 44% of the strains (Tables 1 and 2). No relationship was observed between these major alleles and the sample type, lifestyle or geographical origin of the strains. The majority of SNPs were predominantly (*rpoB*, *aroC* and *gap*) or exclusively (*recA* and *dnaK*) synonymous (Table 3). Loci *omp25* et *trpE* included the majority of nonsynonymous mutations observed (9 and 8, respectively) with a high ratio of non-synonymous to synonymous SNPs. The non-synonymous mutations did not correspond to any premature stop codon.

**Table 3:** Sequence analysis of the seven loci.

Locus (sequence length)	Number of alleles	Number of polymorphic sites (%)	Genetic diversity (h)	Number of non-synonymous codons	dN^a^	dS^b^	dN/dS
*dnaK* (534 pb)	15	19 (3.6%)	0.8293	0	0.000	-	-
*recA* (490 pb)	13	28 (5,7%)	0.7990	0	0.000	-	-
*rpoB* (501 pb)	15	37 (7,4%)	0.7942	1	0.0026	0.0677	0.0384
*aroC* (433 pb)	18	41 (9.5%)	0.7784	2	0.0031	0.1259	0.0246
*omp25* (390 pb)	15	27 (6.9%)	0.6322	9	0.0118	0.0775	0.1522
*trpE* (564 pb)	16	40 (7.1%)	0.8135	8	0.0060	0.0560	0.1071
*gap* (578 pb)	14	35 (6.0%)	0.8101	2	0.0034	0.0575	0.0591

^a^ dN = non-synonymous substitutions per non-synonymous site.

^b^ dS = synonymous substitutions per synonymous site.

### Population structure

The 65 strains studied grouped in 40 sequences types (STs) (Tables 1 and 2). Twenty-nine of them (72%) were identified only once suggesting an overall high level of genetic diversity among the studied population (Tables 1 and 2). The largest STs were ST68 and ST74 (8 and 5 isolates, respectively), grouping 20% of the studied strains. In each of these STs, most strains appeared epidemiologically unrelated, i.e., they were isolated over large periods of time and from geographically distant places. Nine other STs contained 2 or 3 strains. All the strains belonging to ST86, ST90, ST91 and ST96 were clinical isolates whereas ST70, ST71, ST76, ST77, ST84 grouped strains from man and environment. With the exception of ST86 (3 clinical strains, 2 of them being from the same patient), the STs comprising more than 2 strains included strains of clinical and environmental origins. The remaining 29 singleton STs corresponded to clinical (n = 24) and environmental (n = 5) strains.

**Figure 2:**
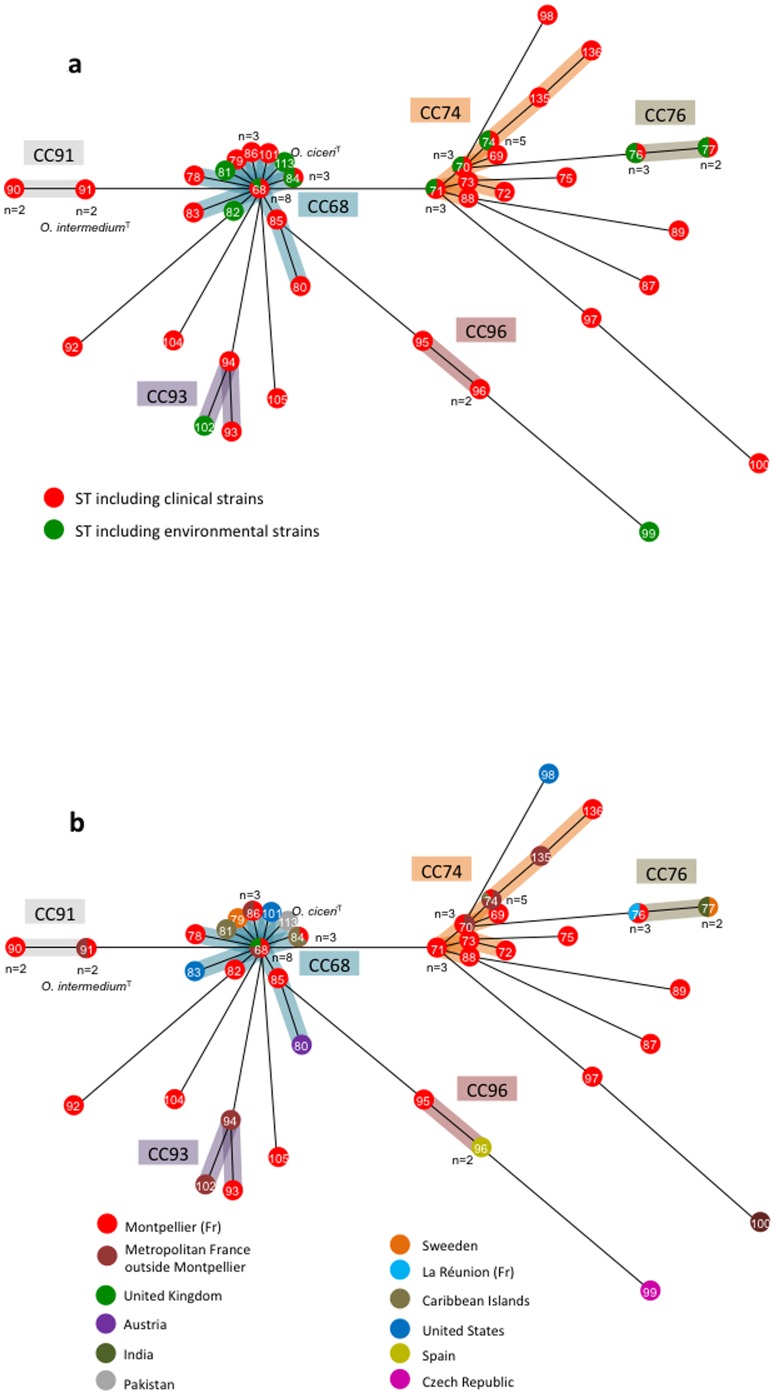
Minimum-Spanning (MS) tree for 64 strains of *O. intermedium* and type strain of *O. ciceri* based on MLST data. The tree was based on the allelic profiles. Each circle corresponds to a sequence type (ST). The number given in the circle corresponds to the ST designation. The number given near the circle corresponds to the number of isolates forming the ST. Shading areas indicate the clonal complexes (CC68, CC74, CC76, CC91, CC93 and CC96). (A) MS tree depending on the clinical (red circles) or environmental origin (green circles) of the strains. (B) MS tree depending on the geographical origin of the strains.

We constructed a minimum-spanning (MS) tree based on clustering of the MLST profiles as a graphic representation of the population structure (Fig. 2a and 2b). In the MS tree, strains formed two major clonal complexes CC68 (23 strains, 12 STs) and CC74 (17 strains, 9 STs), as well as four minor complexes, CC76 (5 strains, 2 STs), CC91 (4 strains, 2 STs), CC93 (3 strains, 3 STs), CC96 (3 strains, 2 STs) and 10 singletons. The type strain of *O. ciceri*, which was the sole representative of the ST113, belonged to the major complex CC68. The locus *rpoB* was the sole to be shared by all strains of the same CC. CC68 strains also shared the same alleles of loci *recA* and *gap* whereas the same allele of *trpE* was found in the CC74. The 4 minor CCs were variable only for loci *aroC* and *omp25*.

No grouping depending on the clinical or environmental origin of the strains was observed (Fig. 2a). The 2 major CCs and the 2 minor CCs CC76 and CC93 included strains isolated from both humans and environment. In contrast, the 2 minor CCs CC91 and CC96 contained only strains of clinical origin (4 and 3 strains, respectively). The population structure did not reflect the strain origin and habitat. For instance, the 4 strains isolated from nematode belonged to 3 STs and 2 CCs.

The geographical origin of strains appeared unrelated to the population structure (Fig. 2b). A high genetic diversity was observed among strains from Montpellier, each CC being represented and 7 strains corresponding to singleton STs. Nearly 90% (16/18) of strains isolated outside Metropolitan France belong to CCs also containing strains from Montpellier, suggesting that the collection studied is representative of the diversity of the species. The CC68 appeared cosmopolitan and included strains isolated from 8 different countries on 3 continents. The CC74 contained only strains isolated in France but in very different regions including Guadeloupe, a Carribean island. The minor CCs were cosmopolitan (CC76 and CC96) or exclusively French (CC91 and CC93).

Two pairs of strains (ADV67-ADV69 and ADV126-127) isolated from the same patient shared the same ST (ST90 and ST86, respectively). By contrast, strains ADV85 and ADV89, isolated from rectal carriage in a same patient more than three months apart and the environmental strains RT148-1P and RT148-2, isolated from the same sample, belonged respectively to different STs.

For clinical strains, there were no relationships between STs and/or CCs and the origin of clinical specimen from which the strain was isolated. Consequently, CCs and STs could not be related to particular body sites and their associated microbiota. Clinical isolates corresponded either to bacterial carriage without related local infection (mainly digestive and axillary carriage) (n = 18) or to the isolation of *O. intermedium* from a body site of infection (n = 31) (Table 1). The “carriage” or “infection” status was unrelated to the strain genetic population structure. Some strains were recovered from sites and fluids that are normaly sterile, such as blood, urine and peritoneal fluid, and again, these strains did not belong to a particular genetic cluster. Similarly, the 4 strains involved in the colonization of the respiratory tract of cystic fibrosis patients belonged to 4 different STs, one being a singleton and the 3 others being distributed in 2 CCs (Tables 1 and 2).

The atypical 16S rDNA insertion was present in all CC68 strains, including *O. ciceri* (Tables 1 and 2). Outside the cosmopolitan CC68, only three singletons (ADV109, Toul55 and CCM7036) and a strain of the CC74 (ADV93) were positive for this insertion. Apart from *O. intermedium*, a database search showed the insertion to be found in *Ochrobactrum daejeonense* (HQ171203) and *Ochrobactrum pituitosum* (AM490609), and outside the genus *Ochrobactrum*, in *Brucella* strains isolated from frogs (HE608873 and HE603360), *Rhizobium* sp. (FM173842), *Paenochrobactrum* sp. (JF804769) and *Phyllobacterium* sp. (GQ183850). The sequences of the insertion were identical for strains belonging to *Ochrobactrum* and *Brucella* sp. but differed by 1 to 6 bp in the 3 other genera.

### Phylogeny and recombination

**Figure 3:**
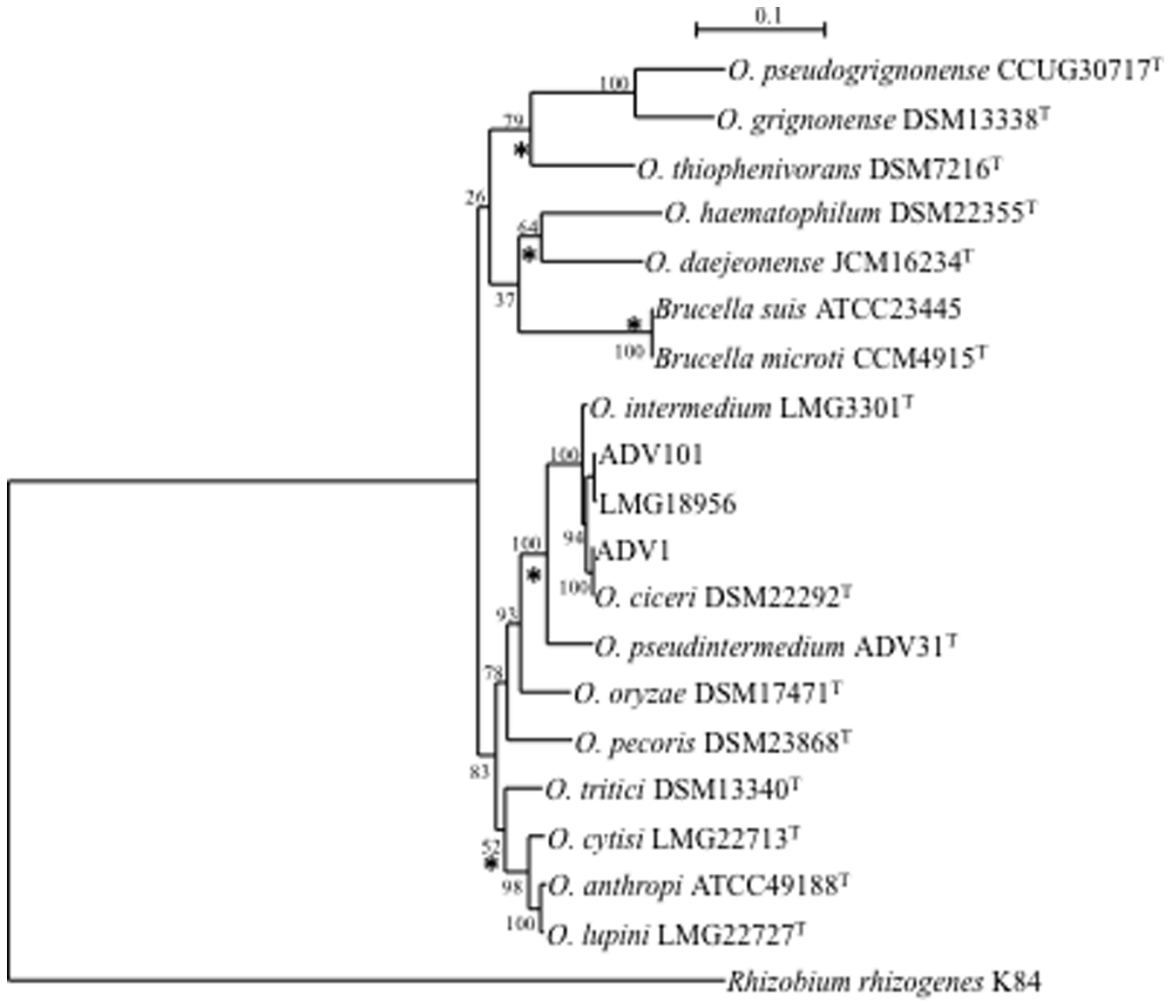
Maximum-Likelihood tree based on concatenated sequences of the seven housekeeping gene fragments of the MSLT scheme indicating the relative placement of type strains of *Ochrobactrum* spp. and 2 strains of *Brucella* spp. The scale bar indicates the number of substitutions per nucleotide position. The numbers at the nodes are support values estimated with 100 bootstrap replicates. *Rhizobium rhizogenes* K84 was used as the outgroup organism. The sequences of *O. intermedium* strains ADV1, ADV101 and LMG18956 that represented the major CC in *O. intermedium* were also included. Asterisks indicate common nodes in Maximum-Likelihood and Neighbor-Joining trees.

Distance and ML phylogenies were reconstructed from concatenated sequences (3480 bp) of the seven loci for 14 type strains of *Ochrobactrum* spp., representatives of major *O. intermedium* clones and 2 strains of *Brucella* spp. In both phylogenies, *O. intermedium* belonged to a robust clade together with *O. anthropi* and 7 other species (Fig. 3). This clade was clearly separated from *Ochrobactrum haematophilum*, *O. daejeonense*, *Ochrobactrum thiophenivorans*, *Ochrobactrum grignonense* and *Ochrobactrum pseudogrignonense* that formed a weak phylogenetic group with *Brucella* spp. in ML phylogeny whereas distance tree placed *Brucella* in outgroup position. Whatever the tree considered, the tree structure did not suggest an ancestral position of *Ochrobactrum* regarding *Brucella* but rather a parallel speciation.

Phylogenetic relationships among *O. intermedium* strains were shown in Figure S1. ML phylogeny confirmed the genetic structure since all CCs corresponded to robust clades. Inside each clade, the relationships between strains reflected their distribution in STs. Of note, the type strain of *O. ciceri* belonged to a *O. intermedium* clade in multilocus phylogeny. ML phylogenies were also reconstructed for each locus (data not shown). The *recA*, *rpoB*, *gap*, *dnaK* and *trpE*-based phylogenies were globally congruent with multilocus phylogeny. In *aroC* and *omp25*-based phylogenies, clade structuration and relationships among strains were not robust due to low phylogenetic signal in the corresponding sequences. Some incongruences between reconstructed phylogenies probably corresponded to genetic exchanges between clades.

**Figure 4:**
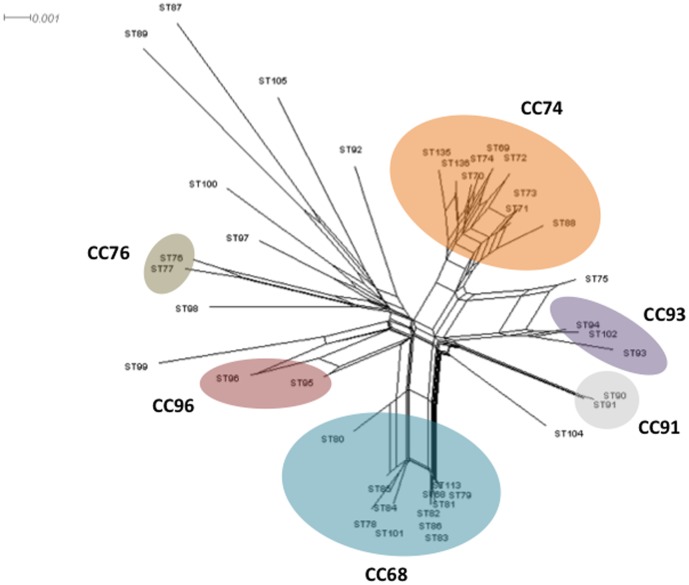
Neighbor-net graph constructed from the concatened sequences of the 40 STs of *O. intermedium* and *O. ciceri* using Splits Tree 4.0. STs are indicated at the branch tips. STs belonging to the same CC are enclosed in an ellipse. A network-like graph indicates recombination events.

Evidence in favor of clonal or recombining population structure can be obtained by assessing the levels of linkage between alleles at different loci by sI_A_ determination. The sI_A_ value is expected to be zero when a population is at linkage equilibrium, i.e., when free recombination occurs. Analyses were carried out using one isolate from each ST in order to minimize bias due to a possible epidemic population structure. sI_A_ was significantly different from zero (sI_A_ = 0.3594; *P* value <10^E^3), suggesting that the recombination rates were low. In contrast, the homoplasy index φ_w_ test, which discriminates between recurrent mutation and recombination, found statistically significant evidence for recombination (*P* value = 0.0). Evidence of recombination was also supported by the Neighbor-Net analysis, which revealed an interconnected network (Fig. 4). Clusters observed were consistent with the population structure determined by MS tree. Recombination events appeared more frequent inside the CCs but also occurred between singletons and CCs, and between singletons. For example, several parallel paths were observed between the singleton ST75 and the CCs CC74 and CC93. All these data suggested that *O. intermedium* displayed a clonal epidemic population structure with recombination events.

### Survey of genome dynamics in *O. intermedium*

**Figure 5:**
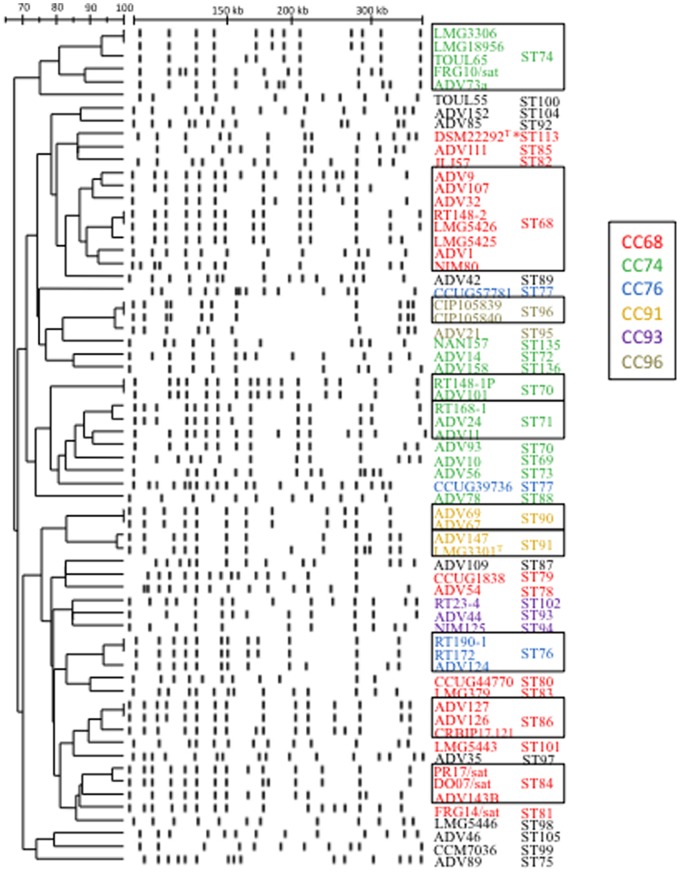
Dendrogram derived from UPGMA cluster analysis of *Spe*I-restricted DNA PFGE patterns of *O. intermedium*/*O. ciceri*. Dice coefficients and approximate fragment sizes are shown at the top of the dendrogram. Clonal complexes (CC) are depicted by a colour code. Sequence types (ST) of the strains were also reported. Frames indicated PFGE clusters grouping strains with the same ST. *; *O. ciceri*.

The genomic DNA of strains was analysed by PFGE after macro-restriction with the endonuclease *Spe*I. The 65 strains showed 58 pulsotypes and nearly 80% (51/65) of strains had a unique pulsotype, indicating the global non-redondancy of the collection (Fig. 5). Seven pairs of strains displaying identical ST shared a pulsotype. Among them, two pairs of strains were isolated from the same patients (ADV67/ADV69 in ST90 and ADV127/ADV126 in ST86). Two other pairs (CIP105839-CIP105840 in ST96 and LMG3306-LMG18956 in ST74) consisted of strains isolated from the same environmental habitat or body site, in the same country but no data on the link between these strains was available from bacterial collections. The three remaining pairs of strains appeared epidemiologically unrelated: LMG5426 and RT148-2 in ST68 were isolated from human urine (UK, before 1983) and water lake (France 2010), respectively; strains ADV101/RT148-1P in ST70 and RT172/RT190-1 in ST76 were respectively isolated from distinct environmental habitats at different dates.

Numerical analysis of the PFGE fingerprints showed an overall conservation of the genomic structure with 67% of pattern similarity in the population. The dendrogram revealed clusters consistent with the multilocus population structure and phylogenies (Fig. 5). Except for ST70 and ST77, strains sharing a ST were grouped into the same PFGE cluster and exhibited more than 80% of pattern similarity. PFGE clustering of strains of the same CC was also observed for CC91 and CC96 (>95% of pattern similarity), and CC93 (>80% of pattern similarity). The two major CCs contained a limited number of PFGE clusters (>80% of pattern similarity): 21 of the 23 strains belonging to CC68 were grouped in 5 PFGE clusters and 15 of the 17 strains belonging to CC74 were grouped in 4 PFGE clusters.

The pulsotypes of the two strains of ST91 differed by only two bands present for strain LMG3301^T^ and absent in strain ADV147. The sizes and sequences were determined from the complete sequence of strain LMG3301^T^ (PRJNA55963) for these two fragments. They corresponded to fragments of 206 and 289 kbp, respectively present on the large and the small chromosome of the bipartite genome of LMG3301^T^. The chromosome size of the two strains was compared by PFGE after macrorestriction with the intronic enzyme I-*Ceu*I. Each of the two chromosomes were cut in two parts by hydrolysis in the 23S rRNA genes. The I-*Ceu*I fragments could be placed on each chromosomes by reference to the complete sequence of strain LMG3301^T^. The fragments corresponding to the small chromosomes had the same size, suggesting that the 289-kbp fragment was not deleted but that chromosomal rearrangement occurred in strain ADV147 (data not show). In contrast, a decrease in size consistent with the loss of a fragment of approximately 200 kbp was observed for the large I-*Ceu*I fragment (2.36 Mbp for LMG3301^T^) of the large chromosome of strain ADV147 without increase in size of the other I-*Ceu*I fragment (240 kb). These results suggested that a genomic deletion occurred during the evolution of this clone. The DNA G+C content of the deleted fragment (59.07%) was similar to the DNA G+C content of the *O. intermedium* LMG3301^T^ genome (57.7%). The deleted fragment corresponded to positions 1734814 to 1941167 of the complete sequence of strain LMG3301^T^ (PRJNA55963) and contained 206 genes. The entire genomic fragment with 206 genes was syntenic in the genome of *O. intermedium* strain M86, the other strain of *O. intermedium* with completely sequenced genome [26]. We considered that these 206 genes were potentially deleted in strain ADV147, even if sporadic translocation of some genes on other genomic contigs could not be excluded. Among them, 32% (65/206) encoded for hypothetical proteins while other genes were mainly involved in transport (16%), metabolism (14%) and regulation (8%). Two percents of genes were pseudogenes and 4% were phage-related sequences. Other minor gene functions were biosynthesis, stress response, chitinase activity, tRNAs, etc. The fonction could not be determined for 16% of these genes. Among the potentially deleted genes, 23% were specific of the *O. intermedium* genome with no significant similarity with any other genomes. Some of the 206 genes were housekeeping genes, such as *trpE*. However, MLSA demonstrated that the *trpE* gene was not deleted in strain ADV147. This result confirmed that some genes, particularly essential ones, might have been translocated to other parts of the genome during a complex genomic rearrangement that included the large deletion.

The gene content of the deleted fragment was further compared with complete genome sequences of related species *O. anthropi* (ATCC 49188^T^) and *Brucella* spp. (*B. microti* CCM4915^T^ and *B. suis* ATCC23445), 77% and 60% of the genes deleted in *O. intermedium* strain ADV147 were present in *O. anthropi* and *Brucella* genomes, respectively. The corresponding genes were distributed in two blocks on the two *Brucella* chromosomes (37% on the small chromosome and 23% on the large one) whereas they were only located on the large chromosome of *O. anthropi*, with a conserved synteny.

The strain ADV1 (ST68) was previously described as having encountered a large deletion of 150 kb and loss of one *rrn* operon during the chronic colonisation of human respiratory tract [9]. The 150-kb deleted fragment was to the smaller band observed in I-*Ceu*I patterns and corresponded to the smaller I-*Ceu*I fragment of the small chromosome. All the 7 other strains belonging to ST68 displayed the 150-kb fragment after I-*Ceu*I restriction and PFGE (Figure S2). However, the strain ADV107 presented a deletion of approximately 100 kb on the 1.8 Mb I-*Ceu*I-restricted fragment corresponding to the large fragment of the small chromosome. Out of the ST68, 6 other CC68 strains displayed large variations in genome size corresponding to 3 indel events of approximately 100 to 400 kb on the small chromosome and 2 indels of approximately 100 and 140 kb on the large chromosome (Figure S2). Genome dynamics was not studied in other CCs such as CC74 and CC76.

In conclusion, the survey of genome size and organisation among the population showed the presence of large deletion events, which appeared as a common mechanism of genomic evolution in the species *O. intermedium*.

## Discussion

Considered as environmental bacteria and human opportunistic pathogens with mild virulence, members of the genus *Ochrobactrum* are increasingly described in human infections (26 publications between January 2005 and October 2013). This genus presented two major traits that worth to be considered for a better understanding of human pathogen emergence : i) its high level of resistance to a large panel of antimicrobial agents and xenobiotics [27]–[29], ii) its membership of a genetically tight clade with *Brucella* spp., a strict and specific anthropozoonotic pathogen [20]. Knowledge about *Ochrobactrum* is therefore of great interest to describe and then to predict the evolution of environmental bacteria towards host pathogenic behaviors.

As observed for numerous EBOPs, the reservoir of *Ochrobactrum* remains unclear and consequently, epidemiology of infections is difficult to assess. The lack of data about *Ochrobactrum* reservoir is mainly due to rare environmental investigations when clinical cases are described but also to difficulties for species identification in the genus. The database survey performed in this study showed that strains of *O. intermedium/O. ciceri* were affiliated to *O. anthropi* in 7.6% of cases including recent publications probably because this species remains the only representative of the genus in several identification system databases. Consequently, the role of *O. intermedium* in human infection remains probably underestimated, as previously suggested [16].

Despite the increasing number of infections due to *O. intermedium*, clones affiliated to this species were not found in human microbiotae [30], except one sequence from normal skin microbiota [23]. Moreover, environmental metagenomics detected scarce *O. intermedium* clones, mainly in polluted or technological environments. Finally, the databanks survey suggests that *O. intermedium* is mostly found in hospitalized patients and in environments modified by human activities suggesting that this minority bacterium is selected by human practices involving the use of xenobiotics such as medicine, agriculture and industry. *O. intermedium* displayed a high level of resistance to several antimicrobial agents, notably to beta lactam antibiotics [16], [31], which represent the most commonly prescribed antimicrobial agents, and antibiotic resistance is frequently associated with resistance to environmental xenobiotics [32], [33]. Altogether, the niche of *O. intermedium* could be described as a human-associated technological niche.

Due to the general lack of specific virulence factors in opportunistic pathogens, population studies gave insights about adaptation to human and/or pathogenic behavior by the detection of specialized clones [7], [34]–[36]. The other human EBOP in the genus *Ochrobactrum*, *O. anthropi*, displayed a human-associated clone suggesting the emergence of human-adapted bacteria from the environmental population [37]. Similar conclusions have been reported for *Agrobacterium tumefaciens*, another EBOP in *Alphaproteobacteria* [38]. The representativeness of the studied population is a pre-requisite to draw such conclusions. Despite a bias in representativeness of *O. intermedium* strains from polluted environments, the genetic diversity of the population studied was high and displayed low redondancy by PFGE typing as well as by examining date and site of strain isolation. We showed that *O. intermedium* displayed a clonal epidemic population structure with recombination events which is a classical structure for EBOP [7], [37], [39]–[41]. CC68 appeared as a cosmopolitan clone for which the local diversity in Montpellier (1999–2010) reflected the mondial diversity (<1983–2005). In contrast, CC74 appeared as a local South French clone including few isolates from other French regions and no isolate from other countries. Whether clonal complexes are cosmopolitan or local, they group human and environmental clones. The clustering of both human and environmental isolates has been observed in CCs and in STs determinated by MLST but also in PFGE fingerprint clusters. The low genetic and genomic diversity in *O. intermedium* cosmopolitan population suggests a clonal success driven by global selective pressures.

Low PFGE polymorphism also evokes genomic synteny that is generally observed in bacteria associated with narrow niches, particularly to eukaryotic host cells, the higher genome stability being reported for intracellular bacteria. For instance, the genetic relatedness assessed by PFGE in the alpha-proteobacteria *Bartonella henselae* is over 80% [42]. In *Brucella*, each species or pathovar displayed a specific PFGE fingerprint [43], [44] suggesting that stable genomes are selected in particular narrow niches represented by the preferential host in the case of *Brucella*. Association with narrow habitat niches is often associated with loss in genomic content compared to free-living relative bacteria [45]. In *O. intermedium* population, we observed indel events leading to decreased genomic size. The indel sequence detailed herein presented a GC% similar to that of the overall genome suggesting that this region was not recently aquired by lateral transfer in LMG3301^T^ but more probably deleted in strain ADV147. Moreover, the presence of most of the homologous genes in the sequence of relative bacteria such as *O. anthropi* and *Brucella* spp. was also in favor of a deletion in strain ADV147. A large deletion event has been previously described in *O. intermedium* [9] and could be suspected for several isolates in our collection particularly in clinical strains of CC91 and CC68. Finally, a previous study showed that megaplasmids, which are frequently described in *Rhizobiales*, were less represented in *O. intermedium* than in *O. anthropi* [10]. Therefore, genome reduction could be considered as a general tendancy in the species *O. intermedium* even if further studies on other CC and environmental strains are required. The bipartite *Brucella* genome appeared very similar to that of *O. intermedium* but showed a genomic size reduced by 1.3 Mb as awaited for a bacterium that lives quasi-exclusively in a narrow cellular niche [46]. Since, phylogeny of *Brucellaceae* did not place *O. intermedium* in an ancestral position regarding *Brucella*, we could consider that genomic reduction is a common theme in *Brucellaceae*.

Genomic deletion in *O. intermedium* ADV1 has been related to the presence of a 46-bp atypical insertion in 16S rDNA [9]. Fourty-seven percent and 41% of environmental and clinical strains of *O. intermedium*, respectively displayed this insertion that could be involved in the tendancy of genomic reduction observed in the population. In addition to *O. intermedium* and its very related phylogenetic neighbour *O. ciceri* [47], identical or similar insertion was also present in *O. daejeonense* [48], *O. pituitosum* [49] and very sporadically in diverse related proteobacteria *rrs*. Of note, atypical mobile and fast-growing *Brucella* strains isolated from frogs [50], [51] displayed similar insertion than *O. intermedium*. These atypical *Brucella* isolates could represent a third evolutionary lineage from the common ancestor of *Brucellaceae*. Among the set of genes used in MLST, *omp25* encodes an outer membrane protein involved in *Brucella* virulence, i.e., invasiveness and intracellular survival [52], and it is noteworthy that a common *omp25* allele was found in 60% of the *O. intermedium* strains, suggesting a particular selective pressure applied on this gene involved in critical envelope properties, like selective permeability. Barquero-Calvo et al. (2009) hypothetized that *Brucellaceae* ancestors carried molecules not readily recognized by innate immunity leading to the emergence of stealthy intracellular pathogens such as *Brucella* [53]. Such hypothesis together with population and genomic data presented here, completes the story of the emergence of pathogenic life-styles among *Brucellaceae* and stresses to study behaviour of *O. intermedium* against eukaryotic cells.

The presence of a cosmopolitan and local clones that presented a genomic stability in a general tendancy of genome reduction suggested that *O. intermedium* is involved in a specialisation process with niche adaptation. This niche is not the eukaryotic cell as observed for *Brucella* because of the large reservoir of *O. intermedium* in man and in various environments but could be a technological niches in which evolution processes are driven by xenobiotics related to human activities in medecine and industry. If such selective pressure is worldwide distributed, it leads to cosmopolitan clone whereas particular pressure could lead to the local emergence of distinct clones. The industrial revolution that opened the anthropocene era [54] could be considered as a global change driving bacterial evolution and emergence of new pathogens as the neolithic revolution did it [55].

## Materials and Methods

### Bacterial strains

A total of 64 strains of *O. intermedium* including 49 clinical and 15 environmental isolates was analyzed (Tables 1 and 2). Clinical strains were sampled over a 39-year period in 6 countries in Europe and North America. Thirty-seven strains were obtained from patients hospitalized in 4 French hospitals in Montpellier, Nîmes (Southern France), Toulouse (Southwestern France) and Nancy (Northeastern France) from 1999 to 2011. Eleven collection strains isolated from man in Europe and the USA were also included, as well as *O. intermedium* LMG 3301^T^. Non-human strains were from diverse origins, including water, soil and invertebrates. They were collected in 6 countries and 4 continents over a 23-year period. The type strain of the species *O. ciceri* was also included. The affiliation of the isolates to *O. intermedium* was assessed by 16S rRNA gene sequencing as previously described [16], [56].

### Ethic statements

No primary human sample materials were used in this study but bacterial isolates from routine clinical diagnostic procedures. This *in vitro* study required neither the agreement of the ethical committee of our institution nor the patient informed consent because it involved only bacterial strains, as stated by the French reglementation.

### Bibliography and BLAST research strategy

The literature search was conducted in March 2013 using Pubmed and the keywords “*Ochrobactrum intermedium*”, cross-references were also considered. When sequence accession numbers were reported in a publication, the affiliation of the sequence(s) to the species *O. intermedium* has been verified. Selection of other *O. intermedium* sequences deposited in databases was performed using the nearly complete 16S rRNA gene of *O. intermedium* strains LMG3301^T^ (AM490623), CCUG44770 (AM114410), LMG18956 (AJ242582), CCUG39736 (AM114408), CCM7036 (AM490631) and of *O. ciceri* strain Ca-34^T^ (DQ647056) using the Megablast program optimized for highly similar sequences for Genbank [57] and the Simrank program for Greengene [58] databases. The sequences having a similarity level of at least 99% with those of previous strains were selected.

### Restriction Fragment Length Polymorphism in Pulsed-Field Gel Electrophoresis (RFLP-PFGE)

Genomic DNA was prepared in agarose plugs as previously described [10] and digested at 37°C with 40 U of *Spe*I (New England Biolabs) or with 1 U of the intronic endonuclease I-*Ceu*I (New England Biolabs). Restriction fragments were separated by PFGE using a CHEF-DRII apparatus (Bio-Rad Laboratories) in a 1% (*Spe*I) or 0.8% (I-*Ceu*I) agarose gel in 0.5X Tris-Borate-EDTA (TBE) buffer at 150 V and at 10°C. Pulse ramps were 5 to 35 s for 35 h followed by 2 to 10 s for 10 h (*Spe*I fragments) or 200 to 300 s for 24 h (I-*Ceu*I fragments). The gels were stained with ethidium bromide and photographed under UV light. *Spe*I-digested DNA from strain *O. intermedium* LMG3301^T^ was loaded on each gel in order to standardize the migration patterns. PFGE bands above 100 kbp were measured with the Mesurim software (http://pedagogie.ac-amiens.fr/svt/info/logiciels/Mesurim2/Telecharge.htm). The bands were scored as present (1) or absent (0) in a binary table, a tolerance of 2% in band position was applied. PFGE patterns were compared by calculation of the Dice correlation coefficient with the FAMD software (http://www.famd.me.uk/) and were clustered into a dendrogram by the unweighted pair group method with the arithmetic average clustering technique.

### Gene amplification and sequencing

Genomic DNA was obtained using the MasterPure™ DNA purification kit (EpiCentre). Seven genes (*dnaK*, *recA*, *rpoB*, *trpE*, *aroC*, *omp25* and *gap*) were amplified using primers and PCR conditions previously described for MLST scheme of *O. anthropi* [37]. PCR products and molecular weight marker (phage phiX DNA digested with *Hae*III, New England Biolabs) were separated in 1.5% (w/v) agarose gels in 0.5X TBE buffer. Amplification products were sequenced in both directions using forward and reverse sequencing primers [37] on an ABI 3730xl automatic sequencer (Cogenics, France). The sequences were deposited to GenBank database with accession numbers KF825086 to KF825540 and KF866307 to KF866369. The primer ins1 was used in association with the universal primer 1492r for specific detection of *rrs* copies carrying a 46-bp atypical insertion previously described [9].

### Phylogeny and decomposition analysis

Gene sequences were codon-aligned using ClustalX after translation with TRANSLATE (http://www.expasy.org). The size of the codon-aligned sequences used for further analyses is indicated in Table 3. Phylogenetic analyses were performed for each of the seven gene sequences and for the manually concatenated sequence. Evolutionary distance was analyzed using Phylip package v3.66 [59] by Neighbor-Joining after distance matrix construction using DNADIST (F84 as substitution model). Bootstrap values were calculated using SEQBOOT and CONSENSE after 1000 reiterations. Maximum-likelihood (ML) analysis was performed using phylogenetic analyses available at http://www.phylogeny.fr [60]. The general time-reversible (GTR) model plus gamma distribution and invariant sites was used as a substitution model. ML bootstrap support was computed after 100 reiterations. The sequences of *O. anthropi* ATCC49188^T^ (PRJNA58921) and/or *Rhizobium rhizogenes* (PRJNA58269) were included in phylogenetic analyses in order to place an artificial tree root.

Along with the phylogenetic reconstruction, we performed a network reconstruction on concatenated data using the Neighbor-net algorithm available in Splitstree4 software [61].

### Multi Locus Sequence Typing (MLST) and multilocus genetics

For each locus, each different allele was assigned to a different arbitrary number using a nonredundant database program available at http://linux.mlst.net/nrdb/nrdb.htm. The combination of allele numbers for each isolate defined the sequence type (ST). A Minimum Spanning (MS) tree was constructed using Prim's algorithm to determine the links among STs (http://www.pubmlst.org). Clonal complexes (CC) included STs that differed by 1 or 2 alleles. The singleton STs corresponded to STs differing from every other ST at 3 or more of the 7 loci.

The LIAN v3.5 program [62] was used to calculate the standardized I_A_ (sI_A_) and to test the null hypothesis of linkage disequilibrium as well as to determine mean genetic diversity (H) and genetic diversity at each locus (h). To detect the presence of recombination events, we also performed the pairwise homoplasy index test, φ_w_ [63], implemented in Splitstree4 [61]. The number of synonymous (dS) and non-synonymous (dN) substitutions per site was determined on codon-aligned sequences using SNAP software [64].

## Supporting Information

Figure S1 **Maximum-Likelihood tree based on concatenated sequences of the seven housekeeping gene fragments of the MSLT scheme indicating the relative placement of 64 strains of** ***O. intermedium*** **and type strain of** ***O. ciceri*** **.** The scale bar indicates the number of substitutions per nucleotide position. The numbers at the nodes are support values estimated with 100 bootstrap replicates. The position of the artificial root (black circle) corresponds to the branching node of the outgroup organism (*O. anthropi* ATCC49188^T^), included in the analysis but not shown on the tree. Clinical strains were noted in blue and environmental strains in green. Clonal complexes (CC) were also reported.
(TIFF)

Figure S2 **PFGE of I-** ***Ceu*** **I-digested genomic DNA from** ***O. intermedium*** **and** ***O. ciceri*** **strains belonging to the CC68.** (A) Lane 1, *Saccharomyces cerevisiae* ladder (Bio-Rad) as molecular size marker with most band sizes in kb; lane 2, ADV1 (ST68); lane 3, ADV107 (ST68); lane 4, ADV32 (ST68); lane 5, ADV9 (ST68); lane 6, Nim80 (ST68); lane 7, LMG5425 (ST68); lane 8, LMG5426 (ST68); lane 9, RT148-2 (ST68); lane 10, ADV143B (ST84). (B) Lane 1, *Saccharomyces cerevisiae* ladder (Bio-Rad) as molecular size marker with most band sizes in kb; lane 2, CCUG1838 (ST79); lane 3, CCUG44770 (ST80); lane 4, LMG379 (ST83); lane 5, *O. ciceri* DSM22292^T^ (ST113); lane 6, LMG5443 (ST101).
(TIFF)

Table S1 **Strains or clones affiliated to** ***O. intermedium*** **/** ***O. ciceri*** **on the basis of 16S rRNA or** ***recA*** **genes sequence analysis in publications (see** **Materials and Methods** **).** Results were sorted by origin. The strains or clones not confirmed to belong to *O. intermedium*/*O. ciceri* were indicated in bold. NA, not applicable. *^a^* 46-bp atypical insertion in *rrs* described by Teyssier et al., 2003 [9].
(XLSX)

Table S2 **Deposited sequences in genetic databases not associated with a publication corresponding to strains or clones of** ***O. intermedium*** **/** ***O. ciceri*** **on the basis of 16S rRNA gene or 16S–23S rRNA intergenic spacer sequence analysis (see** **Materials and Methods** **).** Results were sorted by origin. The strains or clones not affiliated to *O. intermedium*/*O. ciceri* were indicated in bold. NA, not applicable. *^a^* 46-bp atypical insertion in *rrs* described by Teyssier et al., 2003 [9].
(XLSX)
