# Supplementary figures and images for: Niches, Population Structure and Genome Reduction in Ochrobactrum intermedium: Clues to Technology-Driven Emergence of Pathogens
Source: PLoS One. 2014 Jan 17;9(1):e83376. doi: 10.1371/journal.pone.0083376 (PMC3894950; doi:10.1371/journal.pone.0083376)

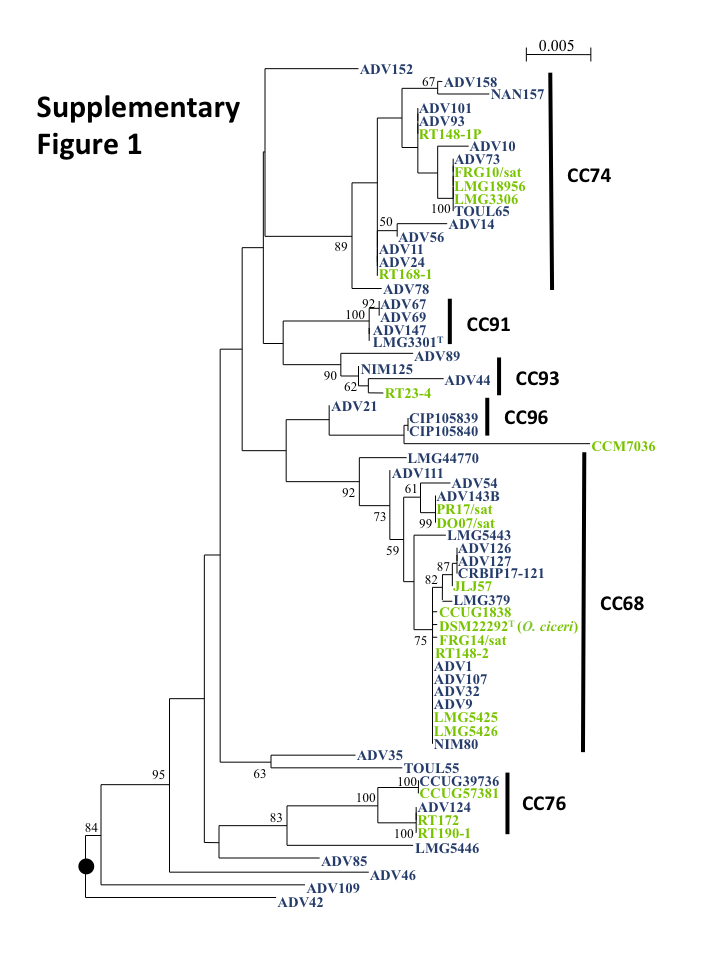

Supplement: Figure S1 — Maximum-Likelihood tree based on concatenated sequences of the seven housekeeping gene fragments of the MSLT scheme indicating the relative placement of 64 strains of O. intermedium and type strain of O. ciceri . The scale bar indicates the number of substitutions per nucleotide position. The numbers at the nodes are support values estimated with 100 bootstrap replicates. The position of the artificial root (black circle) corresponds to the branching node of the outgroup organism (O. anthropi ATCC49188T), included in the analysis but not shown on the tree. Clinical strains were noted in blue and environmental strains in green. Clonal complexes (CC) were also reported. (TIFF) [file pone.0083376.s001.tiff]
